# The Chaperone Balance Hypothesis: The Importance of the Extracellular to Intracellular HSP70 Ratio to Inflammation-Driven Type 2 Diabetes, the Effect of Exercise, and the Implications for Clinical Management

**DOI:** 10.1155/2015/249205

**Published:** 2015-02-26

**Authors:** Mauricio Krause, Thiago Gomes Heck, Aline Bittencourt, Sofia Pizzato Scomazzon, Philip Newsholme, Rui Curi, Paulo Ivo Homem de Bittencourt

**Affiliations:** ^1^Laboratory of Cellular Physiology, Department of Physiology, Institute of Basic Health Sciences, Federal University of Rio Grande do Sul, 90050-170 Porto Alegre, RS, Brazil; ^2^National Institute of Science and Technology in Hormones and Women's Health (INCT-HSM), 90035-003 Porto Alegre, RS, Brazil; ^3^Department of Life Sciences, Regional University of Northwestern Rio Grande do Sul State (UNIJUÍ), 98700-000 Ijuí, RS, Brazil; ^4^School of Biomedical Sciences, CHIRI Biosciences, Curtin University, Perth, WA 6845, Australia; ^5^Department of Physiology and Biophysics, Institute of Biomedical Sciences, University of São Paulo, São Paulo, SP, Brazil

## Abstract

Recent evidence shows divergence between the concentrations of extracellular 70 kDa heat shock protein [eHSP70] and its intracellular concentrations [iHSP70] in people with type 2 diabetes (T2DM). A vital aspect regarding HSP70 physiology is its versatility to induce antagonistic actions, depending on the location of the protein. For example, iHSP70 exerts a powerful anti-inflammatory effect, while eHSP70 activates proinflammatory pathways. Increased eHSP70 is associated with inflammatory and oxidative stress conditions, whereas decreased iHSP70 levels are related to insulin resistance in skeletal muscle. Serum eHSP70 concentrations are positively correlated with markers of inflammation, such as C-reactive protein, monocyte count, and TNF-*α*, while strategies to enhance iHSP70 (e.g., heat treatment, chemical HSP70 inducers or coinducers, and physical exercise) are capable of reducing the inflammatory profile and the insulin resistance state. Here, we present recent findings suggesting that imbalances in the HSP70 status, described by the [eHSP70]/[iHSP70] ratio, may be determinant to trigger a chronic proinflammatory state that leads to insulin resistance and T2DM development. This led us to hypothesize that changes in this ratio value could be used as a biomarker for the management of the inflammatory response in insulin resistance and diabetes.

## 1. Introduction

Heat shock proteins (HSPs) are considered part of a family of proteins known as “stress proteins” since their expression is induced by a wide range of stressors, such as oxidative stress [1], thermal stress [2], ischemia [3], exercise [1], metabolic stress [4], and many others. The 72 kDa member of the 70 kDa family of heat shock proteins, HSP70 (or HSPA, encoded by the HSPA1A gene in humans), is inducible during cell stress. It is the most abundant of all HSPs, accounting for 1-2% of cellular protein [5], and is plentiful in skeletal muscle [6]. As molecular chaperones, the intracellular HSP70 proteins (iHSP70) can interact with other proteins (unfolded, in nonnative state and/or stress-denatured conformations) to avoid inappropriate interactions, formation of protein aggregates, and degradation of damaged proteins, as well as helping the correct refolding of nascent proteins [6]. Other functions include protein translocation [7], antiapoptosis [8], and anti-inflammatory responses [9, 10]. More recently, the HSP roles have been expanded to include control of cell signaling [11], modulation of immune response [12], and modulation of chronic disease conditions [13] such as diabetes, obesity, and insulin resistance [14, 15].

The heat shock response is regulated by a family of heat shock transcription factors (HSFs) composed of four members (HSF 1–4), which are maintained in an inactive monomeric form during nonstimulated conditions [16]. HSF-1 is a primary regulator of heat shock response in mammalian cells and a low concentration of it has been associated with a number of human pathologies including T2DM [17] and obesity-related fatty liver disease [18]. HSF-1 activation is a multistep mechanism that involves its phosphorylation, trimerization, nuclear translocation, and DNA binding to heat shock elements (HSE) located at the promoter regions of targeted heat shock genes [15]; nevertheless, HSF-1 activation can be negatively regulated by posttranscriptional modification, such as phosphorylation in specific serine residues and phosphorylation-dependent sumoylation [19].

Heat shock proteins were long thought to be exclusive cytoplasmic proteins with functions restricted to the intracellular compartment. However, an increasing number of observations have indicated that they may be released into the extracellular space (eHSP70) having a wide variety of effects on other cells [20]. eHSP70 function is, in general, associated with the activation of the immune system [21]. For example, eHSP70 has been reported to stimulate neutrophil microbicidal capacity [22] and chemotaxis [23] and recruitment of natural killer (NK) cells [24] as well as cytokine production by immune cells [12, 25]. In addition, eHSP70 was recently hypothesized to be involved in the inducement of neural cell protection under stress conditions [15].

An intriguing aspect of HSP70 physiology is its versatility to induce antagonistic actions, depending on the location of the protein [17]. For example, iHSP70 exerts a powerful anti-inflammatory effect, while eHSP70 has the opposite role, inducing the activation of several proinflammatory pathways. In fact, chronic exposure to eHSP70 [26] induces the activation of several proinflammatory pathways probably via binding to membrane Toll-like receptors (see below) although eHSP70-peptides have also been shown to act as anti-inflammatory and immunosuppressive factors after internalization and antigen processing (see Borges et al., 2012, for review [27]).

iHSP70 exerts its anti-inflammatory effect through the interaction with the nuclear factor *κ*B (NF-*κ*B), blocking its activation [28]. NF-*κ*B is a ubiquitous transcription factor originally discovered in B-lymphocytes that is essential for arming inflammatory responses to a variety of signals, immune function, endothelial cell activation, and the control of cell growth [29]. iHSP70 hampers NF-*κ*B activation at several levels, by impeding the phosphorylation of inhibitor of *κ*B (I*κ*Bs) [30], by directly binding to I*κ*B kinase gamma (IKK*γ*) [31], which will result in continued binding (and inactivation of NF-*κ*B) thus inhibiting downstream inflammatory signals. This is corroborated by the finding that iHSP70 binds with liver NF-*κ*B/I*κ*B complex in the cytosol thus hindering transcription of TNF*α* and inducible nitric oxide synthase (NOS2) genes [31] which are activated via a NF-*κ*B dependent mechanism. Stress-induced elevations in iHSP70 inhibit c-Jun N-terminal kinase- (JNK-) dependent signal transduction hence promoting cell survival [32].

iHSP70 affects apoptosis at various levels. It can inhibit caspase activation by interfering with Apaf-1 and prevent the recruitment of procaspase-9 to the apoptosome [33]. HSP70 also increases Bcl-2 expression and inhibits cytochrome C release [34]. Overexpression of HSP70 in lymphoid tumor cell lines inhibited apoptosis by attenuation of caspase activation [35]. The antiapoptotic effects of HSP70 have been reported for mouse brain tissue, where HSP70 overexpression resulted in decreased infarct sizes, improved neurological deficits, and fewer apoptotic cells (determined by reduced DNA laddering) after middle cerebral artery occlusion [36]. HSP70s have also been shown to decrease oxidative stress so that they are part of the intracellular antioxidant machinery, making iHSP70 even more important for the inhibition of apoptosis and inflammation [37]. Cyclopentenone prostaglandins (cp-PGs), which under certain circumstances may induce HSP70 expression, are consequently powerful anti-inflammatory autacoids [38–40].

The interplay between iHSP70 and proinflammatory cytokines at gene regulatory level has also been reported. The promoter region of TNF*α* gene contains an HSF1 binding site that represses TNF*α* transcription, and thus loss of this repressor results in sustained expression of TNF*α* [41]; thus the HSF1 knockout is associated with a chronic elevation of TNF*α* levels and increased susceptibility to endotoxin challenge [42]. Regulation of such a network in the opposite directions has also been demonstrated: TNF*α* may transiently repress HSF1 activation [43]. Furthermore, JNK1 was unequivocally demonstrated to phosphorylate HSF1 in its regulatory domain causing suppression of HSF1 transcribing activity [44] while HSP70 prevented Bax activation both by inhibiting the JNK/Bim pathway and by interacting with Bax in UV-induced apoptosis [45]. Altogether, the above findings explain why the induction of HSP72 (HSPA1A)* in vitro* (by heat shock or HSP72 transgene overexpression) reduces the expression of inflammatory genes such as TNF*α*, IL-1, IL-12, IL-10, and IL-18 [46].

In contrast to the above findings, eHSP70 proinflammatory actions have been demonstrated to be mediated by MyD88/IRAK/NF-*κ*B signal transduction pathway after both Toll-like receptor 2 (TLR2) and TLR4 binding, in a CD14-dependent manner [47, 48], thus promoting innate immune activation [49]. Due to the antagonistic actions of the heat shock proteins within the course of an inflammatory response, it is reasonable to hypothesize that the balance between eHSP70 and iHSP70 might determine the outcome—either the induction or the attenuation of inflammation. Since low-grade inflammation is involved in several chronic diseases [17], the management of HSP70 expression and its location can be crucial for the control of inflammatory-related conditions, such as T2DM. We herein suggest that the ratio of the extracellular medium HSP70 concentration to intracellular HSP70 contents (eHSP70/iHSP70) can determine the progress of insulin resistance and the progression of T2DM.

## 2. Obesity, Low-Grade Inflammation, and Insulin Resistance

The incidence of T2DM has increased dramatically over the last fifty years and this is clearly associated with growing rates of obesity [50] and physical inactivity. Obesity is linked to a chronic proinflammatory state, since adipose tissue expansion and adipose associated immune cell activation result in the release of several cytokines, such as TNF*α*, which leads to the activation of serine threonine kinases JNKs and IKK [14]. It is known that both JNK and IKK phosphorylate insulin receptor (IR) substrate-1 (IRS-1) on Ser-307, leading to the inactivation of the insulin receptor downstream response [14]. Also, chronic activation of IKK has been reported in diabetic patients, while a reduction in IKK activity prevents the development of insulin resistance* in vitro* and* in vivo* [51]. In addition, lipid oversupply and hyperglycemia can lead to increased deposition of lipid species such as diacylglycerols and ceramides, which can also activate JNK and IKK in liver and/or skeletal muscle, leading to insulin resistance [52], causing sustained hyperglycemia and hyperlipemia.

Hyperglycemia* per se* is also known to be involved in inflammation and diabetes-associated vascular complications arising from reactive oxygen species generation and action [53, 54]. Chronic hyperglycemia induces the production of reactive oxygen species (ROS) [55], leading to enhancement of protein oxidation, DNA oxidation, and lipid peroxidation. The free radical gas nitric oxide (NO^∙^) also plays a role in the insulin resistant state generated by proinflammatory cytokines. NO^∙^ is synthesized at high rates by the inducible form of nitric oxide synthase (iNOS, encoded by the NOS-2 gene) which plays a significant role in cell damage associated with obesity and T2DM. Interestingly, a physiological concentration of this free radical is required to stimulate necessary functions such as muscle GLUT4 expression/translocation [56] and insulin secretion by *β*-cells [57]. However, at high concentrations, NO^∙^ compromises insulin-stimulated glucose transport in skeletal muscle and can also be toxic to *β*-cells inducing death. Accordingly, acute treatment with NO^∙^ donors results in a reduction of insulin-stimulated glucose uptake and glycogen synthesis in isolated soleus muscle, inducing decreased IR*β* and IRS-1 activity.* In vivo*, this treatment also promotes insulin resistance in healthy animals by the reduction of IRS-1 levels. Obese* ob/ob* mice or rats submitted to high fat diet (HFD) have shown enhanced NOS-2 expression associated with insulin receptor and Akt S-nitrosylation, which can be dismissed by rosiglitazone treatment by virtue of its NOS-2 expression inhibiting activity [58].

Expanded adipose tissue triggers the release of interleukin-6 (IL-6) in obese subjects that is associated with alterations in glucose uptake by the skeletal muscle [59]. Nevertheless, it is possible that there is a dual role of serum IL-6 on glucose metabolism, probably related to the exposure time of and concentration of IL-6. Accordingly, acute IL-6 treatment may increase glucose uptake in C2C12 myotubes by stimulating AMP-activated protein kinase (AMPK) in a serine/threonine kinase 11- (LKB1-) dependent pathway, which induces downstream AS160 activation [60], while IL-6 may induce a modest increase in the glucose infusion rate after 4 h of hyperinsulinemic-euglycemic clamp in mice [61]. Moderate doses of IL-6 stimulate basal and insulin-stimulated glucose uptake in L6 myotubes and 3T3 cells line after 2 h [61]. In addition, physiological concentrations of IL-6 were reported to stimulate insulin secretion by isolated pancreatic islets and BRIN-BD11 clonal *β*-cells through AMPK activation [62]. However, chronic treatment (24 h) with this cytokine has been demonstrated to be able to cause insulin resistance in C2C12 murine myotube cell line, due to impairment of insulin signaling (IRS/AKT cascades) in a JNK1/2-dependent manner [60].

Finally, obesity is often associated with a vicious cycle in which adipose tissue expansion increases the levels of free fatty acids (FFA) and proinflammatory cytokines in the circulation, which together with hyperglycemia and altered lipoprotein profiles increase the synthesis and accumulation of intramyocellular triglycerides (IMCT) [63]. Sedentary behavior and aging are conditions related to a decreased mobilization of the IMCT resulting in an increased synthesis of toxic fatty-acid-delivered metabolites (FADM). These metabolites cause, in turn, an elevation in the production of reactive oxygen and nitrogen species (ROS and RNS), resulting in oxidative/nitrosative stress, mitochondrial dysfunction, and the activation of stress associated transcription factors, such as NF-*κ*B, which is followed by increased production and release of proinflammatory cytokines (e.g., TNF*α*). TNF*α* is a major driver of insulin resistance in skeletal muscle and, in addition, it can also induce activation of stress signals in pancreatic *β*-cells, leading to mitochondrial dysfunction that culminates in cell failure and death [64].

## 3. Role of iHSP70 in Insulin Sensitivity

It has been reported that iHSP72 mRNA levels are decreased in skeletal muscle of T2DM patients which is correlated with the “status” of insulin resistance [65], whereas heat shock-like therapies (i.e., whole body warming, transgenic overexpression, or pharmacological mechanisms to elevate HSPA1A protein expression) protect against high-fat-diet- and obesity-induced hyperglycemia, hyperinsulinemia, glucose intolerance, and insulin resistance [14, 65, 66]. Although the underlying mechanism(s) culminating in lower iHSP70 expression in T2DM individuals are not fully understood, the observed reductions are likely to be a result of the concerted contribution of (i) reduction in the rate of HSP70 protein synthesis due to attenuation of initiation and elongation phases of translation, (ii) suppression of HSF-1 activation and binding to HSE via an increase in glycogen synthase kinase-3*β* activity (GSK-3*β*) as previously suggested [67], and (iii) decreased HSF1 expression. In this regard, it has been recently proposed that long-term inflammatory stimuli emanating from the adipose tissue of obese individuals could repress HSF-1/iHSP70 axis because continuous activation of inflammasome NLRP3 may lead to a state of cellular senescence which abolishes the expression and activity of HSF-1 [68]. In line with this is the observation that the expression of both HSF-1 and iHSP70 in adipose tissue and the liver of type III obese patients is dramatically reduced while the activation of JNKs is reciprocally enhanced in the same tissues [18].

As discussed above, the anti-inflammatory effect of iHSP70 is attributed mainly to its capacity of interaction with NF-*κ*B. In fact, IKK-*β* and NF-*κ*B activity have been found to be increased in different obese experimental models. Chronic activation of IKK has been reported in diabetic patients, while a reduction in IKK activity prevents the development of insulin resistance* in vitro* and* in vivo* [51]. High-fat, high-carbohydrate intake may result in oxidative stress and consequent NF-*κ*B activation in obese subjects [69]. On the other hand, NF-*κ*B DNA binding is suppressed after heat shock [70], and upregulation of HSPA1A can negatively affect NF-*κ*B activity in skeletal muscle [71]. In addition, overexpression of HSPA1A can restrict NO production and release by transfected cells in a mechanism dependent on NF-*κ*B DNA binding and subsequent iNOS gene expression [70].

Heat treatment is capable of inducing HSPA1A expression in several tissues, preventing various obesity-elicited metabolic effects at molecular level, leading to improvement of glucose tolerance, insulin-stimulated glucose transport, and insulin signaling accompanied by the reduction in JNK and IKK*β* activities in skeletal muscle [72] and liver [14] of HFD mice, which is almost completely abolished in transgenic HSPA1A^+/+^ mice [14]. Furthermore, iHSP70 expression has been shown to decrease JNK activity, irrespective of stress stimulus [73]. This is crucial for insulin sensitivity, since enhanced rate of JNK phosphorylation is associated with glucose intolerance and insulin resistance in skeletal muscle of obese mice, an effect which may be attenuated by long-term (16-week) heat treatment (41.5°C) and is also observed in transgenic HSPA1A^+/+^ mice [14]. Heat treatment also induced improvement of mitochondrial function, increasing citrate synthase and *β*-hydroxyacyl-CoA-dehydrogenase (*β*-HAD) activity in soleus and extensor digitorum longus (EDL) muscle [14]. Interestingly, a 12-week period of heat treatment is unable to change fasting blood glucose of obese rats, but it does attenuate insulin levels and decrease whole body glucose clearance induced by HFD [72].

In obese HFD animals, phosphorylation of IRS1 in Tyr612 and the consequent downstream phosphorylation of Akt in Ser473 and activation of AS160 have been found to be reduced in the skeletal muscle, while heat treatment reverted this response in a process associated with HSPA1A expression [72]. Moreover, JNK inhibition has been demonstrated to rapidly occur in a dose-dependent manner in heat-shocked NIH 3T3 fibroblasts via interaction between JNK1 and HSPA1A [74]. Finally, studies have demonstrated that HSP70 is able to bind to the insulin receptor enhancing its recycling rate after heat shock [75, 76], which suggests that heat shock proteins may have direct influence upon insulin receptor function and activity.

## 4. Role of eHSP70 in Insulin Resistance and ***β***-Cell Dysfunction

In contrast to its intracellular proinsulin signaling and anti-inflammatory effect, extracellular HSP70 (eHSP70) when chronic elevated is associated with inflammatory conditions, including T2DM [17]. Other cells (e.g., lymphocytes, macrophages, epithelial cells, dendritic cells, neuronal cells, and hepatocytes) have been reported to release HSP70 proteins via (i) active mechanisms, such as vesicular secretion (classical pathway in rest conditions), (ii) lipid rafts, or (iii) exosomes [77]. Once in the bloodstream, eHSP70 may act as a paracrine factor [77].

With respect to the extracellular compartment, eHSP70 can bind to TLR2 and TLR4 in a variety of cells, [77] leading to the activation of proinflammatory pathways via MyD88 and TIRAP that signal downstream to NF-*κ*B via IRAK4, TRAF6, and IKK and inducing JNK activation via MEKK4/7 [49, 78], although high-affinity binding of eHSP70 to other surface receptors has also been described [79]. The signal triggered by eHSP70 promotes typical immunoinflammatory responses directed to the combat of infections and bacterial infiltration through the production and release of NO^∙^ and proinflammatory cytokines, such as TNF*α* and IL1*β* [80]. Moreover, eHSP70 responses are positively associated with classical inflammatory parameters such as CRP, fibrinogen, and monocyte counts [81] being commonly found in clinical situations, in which a danger signalization to immune system must be required [77]. Indeed, increased serum HSP70 has been reported in chronic and age-related diseases [82–84].

Interestingly, during conditions in which individuals are chronically exposed to elevated eHSP70 levels, changes in the iHSP70 content are also observed [14, 65]. In fact, in T2DM iHSP70 is reduced in insulin-dependent tissues such as skeletal muscle and adipose tissue [14, 17, 65] while the eHSP70 is elevated. This profile is commonly found in T2DM, where obesity is an aggravating factor for the undesirable high eHSP70/iHSP70 ratio [17]. In addition, serum HSP70 levels were found to be higher in long-term (>5 years) T2DM patients as compared to newly diagnosed ones [85].

High eHSP70-mediated stimulation of TLR2/4 may severely jeopardize insulin signaling. Accordingly, TLR2/4-dependent activation of JNKs promotes phosphorylation of IRS-1 at Ser307 in rodents (equivalent to Ser312 in humans) leading to inhibition of Akt activation [86] and, consequently, to a reduced glucose uptake by sensitive tissues and to a state of resistance to insulin action. Moreover, it has been shown that TLR2 is central to palmitate-induced insulin resistance [87], via JNK-mediated phosphorylation of IRS1/2 [88]. On the other hand, loss-of-function mutation in TLR4 prevents HFD-induced obesity and insulin resistance [89].

Ser307 phosphorylation of rodent IRS-1 may also be elicited by inflammatory cytokines via IKK, in a process that can be inhibited by cp-PGs [90], which are powerful anti-inflammatory autacoids possessing iHSP70-inducing capacity [40]. Indeed, inhibitory Ser307 phosphorylation of IRS-1 is a physiological mechanism of feedback inhibition of insulin signal that is under the control of both insulin/IGF1 and inflammatory cytokines, though via different downstream pathways [91]. TLR4 expression and signaling dependent on eHSP70 is increased in obese and T2DM subjects, an effect that can explain the high basal rate of MAPK phosphorylation and NF-*κ*B activation found in these patients [92–95]. The above findings help to explain why inhibition or absence of TLR4 confers protection against insulin resistance in skeletal muscle [96], adipose tissue, and liver [26, 97].

Finally, as recently found, eHSP70 is positively correlated with insulin resistance and inflammation in elderly people. This may indicate a role for eHSP70 in impairment of insulin signaling in the skeletal muscle that occurs with advanced age and in T2DM [98]. In addition, the same group has shown that chronic exposure of *β*-cells and islets to increased concentrations of eHSP70 results in *β*-cell death and altered cell bioenergetics, a phenomenon that, apparently, is mediated through TLR-2 and TLR-4 activation [98]. Since, in T1DM there is a dramatic increase in eHSP70 and in T2DM and aging there is a slow chronic increase in the concentration of this protein, we educe that chronic exposure of pancreatic *β*-cells to eHSP70 may lead to *β*-cell failure and loss of functional integrity* in vivo*.

## 5. The eHSP70 to iHSP70 Ratio: The Chaperone Balance Hypothesis

Based on the previous discussion, while iHSP70 is clearly protective, antiapoptotic, anti-inflammatory, and associated with normal insulin sensitivity, eHSP70 is related to a proinflammatory response, decreased expression of the anti-inflammatory iHSP70, and reduced insulin sensitivity. Because of this, we suggest that the ratio of compartmental distributions of HSP70 between extra- and intracellular locations may determine the outcome of the inflammation and its associated insulin resistance.

In a recent study, our group observed that the ratio between plasma eHSP70 and iHSP70 in lymphocytes from rats submitted to different loads of acute exercise can indicate the inflammatory status (Heck et al., manuscript in preparation). Accordingly, assuming the ratio *R* = [eHSP70]/[[iHSP70] = 1 for the controls (resting, unstimulated), moderate exercise produces a shift in *R* to up to* ca.* 5, which is paralleled by an elevation in inflammatory markers and stimulation of cell proliferation. *R* values higher than 5 denote an exacerbated proinflammatory response. Conversely, *R* values between 0 and 1 indicate a predominantly anti-inflammatory status. Thus, changes in the ratio between extra- and intracellular HSP70 emerge as a potentially new biomarker for inflammation and as a very sensitive indicator of inflammatory status.

We applied this simple mathematical calculation to published data from studies elsewhere. For instance, Yang and collaborators have investigated the correlation between the level of exposure to pollution and eHSP70 in steel workers [99]. From the data obtained in this study, we calculated *R* (plasma to lymphocyte ratio) as 5.5, 6.5, and 8.8, respectively, for low, moderate, and high exposure, as compared to controls (*R* = 1.0). We applied the same calculation to the data by Rodrigues-Krause and colleagues' obesity-diabetes study [17], in which HSP70 was investigated in healthy obese (considered the controls herein), nonobese T2DM and in obese T2DM patients. In this case, settling controls as *R* = 1.0, we get 1.8 for T2DM and 6.0 for obese T2DM patients in which inflammatory unbalance was found. Although healthy lean subjects had not been evaluated in this work, it is likely that such *R* marks should be even higher if *R* were taken in comparison with such controls. Additionally, changes in *R* calculation seem to be valid during heat exposure as *R* values correlated with the heat exposure of peripheral blood mononuclear cells to different temperatures within a physiological range. Accordingly, [100] heat-shocked cells concomitantly measured HSP70 at 37°C (*R* = 1.00), 39°C (*R* = 1.45), 42°C (*R* = 0.65), and 43°C (*R* = 0.48). Interestingly, at 45°C, in which cells are known to trigger JNK-dependent prosurvival inflammatory pathways [74], *R* value was calculated as 1.97, confirming proinflammatory expectations. Moreover, in a combined protocol of exercise training (60 min during 11 days in a treadmill, 1.69–2.20 m·s^−1^, 1% grade) and heat acclimation (rectal temperature elevation by 1°C for the duration of the exercise sessions at 40°C room temperature), [101] have observed, in human volunteers, HSPA1A alterations (in plasma and total leukocytes) that, after conversion to *R* values, furnish the following picture: before training, *R* values were 1.11 at rest and 0.51 two days after the primary test; after training and acclimation, *R* values were 0.22 at rest and 0.27 two days after the last training session. These values can be explained by the fact that eHSP70 plasma contents raised by* ca.* 50% in untrained individuals evaluated 48 h after the priming test, while under the same circumstances, iHSP70 was found to be 2.9-fold the remaining values. On the other hand, exercise training combined with heat acclimation evoked a 4.2-fold enhancement in intracellular HSPA1A contents (hence, an anti-inflammatory response) which remained unaltered 48 h after the last session, while eHSP70 did not change any more, remaining near the rest of values observed in untrained volunteers.

**Figure 1:**
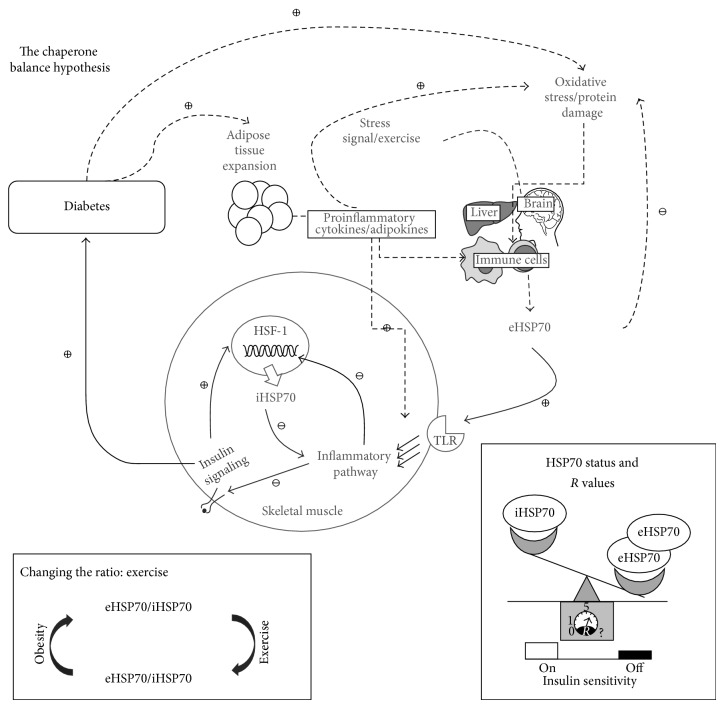
The chaperone balance. Adipose tissue expansion leads to chronic release of proinflammatory cytokines and adipokines. The low-grade inflammation can induce (i) activation of NF-*κ*B-dependent inflammatory pathways leading to the blockage of iHSP70 and to insulin resistance, (ii) release of proinflammatory eHSP70 chronically from immune cells, and (iii) ROS and RNS, oxidative/nitrosative stress that leads to protein damage and denaturation. eHSP70 is increased as a danger signal and to combat the plasma oxidative damage; however, when chronically elevated, it (i) induces further immune activation and proinflammatory response and (ii) activates TLR and the inflammatory pathway leading to the reduction of HSF-1 activation and eventually to reduced iHSP70. Lower iHSP70 causes (i) reduced insulin sensitivity, (ii) intensification of the NF-*κ*B activation and inflammation, and (iii) reduced antioxidant, antiapoptotic, and anti-inflammatory capacity. The long-term insulin resistance determines the onset of diabetes, completing this positive feedback mechanism. Lower panels: when the eHSP70/iHSP70 ratio chronically changes in favor of eHSP70, the “insulin sensitivity button” is switched off and *R* values ([eHSP70]/[iHSP70]) rise; exercise induces iHSP70 expression while the release of eHSP70 responds in an opposite manner. *R* values between 0 and 1 indicate an anti-inflammatory status and between 1 and 5 indicate an optimum immunoinflammatory surveillance status, while *R* values above 5 suggest an undesirable chronic proinflammatory status.

The above observations led us to postulate that *R* values and, particularly, changes in *R* values could be used as a predictor gauge for the inflammatory response that culminates in insulin resistance and diabetes, despite the method used to assess intra- and extracellular HSP70 contents. Notwithstanding, *R* values may be also useful for application in several other inflammatory diseases and conditions besides being of value in controlling exercise impact over inflammatory status. Furthermore, since eHSP70 and iHSP70 are directly related to insulin sensitivity, *R* value application in diabetes appears to be straightforward.  Figure 1 summarizes this hypothesis.

## 6. Changing* R* Values: The Role of Physical Exercise

As discussed above, eHSP70 to iHSP70 ratio (represented by *R* values) may determine the fate of the insulin sensitivity towards either its improvement (lower ratio) or its reduction/impairment (higher ratio). In this regard, strategies capable of changing the HSP70 contents, in the intra- or extracellular space, or both compartments, are likely to be used as a therapeutic strategy for the prevention or treatment of T2DM and its efficiency could be precisely followed by the assessment of *R* values during the course of such treatment. In this sense, physical exercise fulfills all the prerequisites: it is a known modulator of eHSP70 (whose levels drop under exercise training) and iHSP70 (which tends to rise under the same circumstances). In other words, although acute exercise bouts signalize a “stressful situation” to all physiological systems (please see Heck et al. 2011 for review [102]) leading to augmented but just momentary eHSP70 plasma levels [103], exercise training or regular physical activity tends to reduce the stressful impact of each exercise session, leading to decreased eHSP70 and enhanced iHSP70 during the course of training. For this reason, exercise can also be used as a tool to decrease or maintain the normal *R* values and, consequently, optimum insulin sensitivity. In fact, improvement of glucose uptake and storage as well as increase in the oxidative capacity of different muscle fibers has been shown to be associated with increased iHSP70 expression in the skeletal muscle [14, 66]. Moreover, physical exercise has been proposed as an alternative strategy for T2DM patient treatment by virtue of its iHSP70 enhancing capacity: patients submitted to acute exercise bouts or moderate training have shown significant increase in muscle HSP70 expression [66, 104], which is directly associated with reduction of insulin resistance [14, 72].

Exercise-induced expression of iHSP70 in skeletal muscle has a major role in restoring muscle metabolic functionality; besides, it provides cytoprotection to damaged cells. Such an iHSP70 inducing ability has been shown as a resultant in different protocols of exercise, including eccentric, concentric not damaging, aerobic, or resistive, all of which are capable of inducing intramuscular HSP70 expression [104–106]. Although time and intensity of the physical effort are determinant factors to increase of intramuscular HSPA1A, its rise may be detectable just 2 h after the onset of an acute exercise session, when HSPA1A mRNA expression peaks [107]. Moreover, exercise-induced iHSP70 presents a time and intensity dependence [108].

Since iHSP70 family members promote the facilitation of protein transport into mitochondria, allowing and improving structural integrity of the organelle during fast energy flow, iHSP70 content has been correlated with an increase in the oxidative capacity of muscle cells. Several studies have demonstrated the relationship between high iHSP70 levels in skeletal muscle and increased activity of mitochondrial enzymes after short training period [109]. On the contrary, decreased mitochondrial function is known to be associated with the accumulation of intramyocyte triglycerides (and its byproducts), insulin resistance, and diabetes. For this additional reason, exercise-induced iHSP70 expression can lead to improvements in metabolite oxidation and, consequently, insulin sensitivity.

As regarded above, HSF-1 is negatively regulated by GSK3*β*, a serine/threonine kinase that phosphorylates this factor on Ser303, keeping it in its inactive form in the cytosol. During acute physical exercise, GSK3*β* activity has been found to be nevertheless 30% decreased in Vastus lateralis muscle, concomitantly with Akt phosphorylation in Ser473 and glycogen synthesis (GS) activation [110]. In addition, a direct relation between iHSP70 and IL-6 has been reported. During physical exercise, IL-6 can be expressed [111] and released [112] by the skeletal muscle and, within extracellular space, binds to the IL-6 receptor in an autocrine action [113]. Interestingly, the “myokine” IL-6 has also been found to induce HSF-1 translocation to the nucleus upregulating heat-induced HSP70 gene, protein expression, and activity in human hepatic cells in a PI3K/Akt/GSK3*β* dependent pathway [114]. On the other hand, absence of IL-6 is associated with decreased expression of HSPA1A in skeletal and cardiac muscle of mice challenged with LPS, although IL-6 seems not to be required for exercise-induced HSP expression [115].

The molecular mechanism(s) underlying the connection between iHSP70 expression and increased energy flow are not clear yet. However, changes in ATP/ADP ratio seem to work as a signal to the activation of different kinases, such as AMPK that is capable of decreasing GSK3*β* activity [116]. AMPK is also regulated by Ca^2+^ during muscle contraction that activates Ca^2+^/calmodulin protein kinase kinase (CaMKK) in an LKB1-dependent pathway [117]. Thus, AMPK activation as result of muscle contraction may act as a stimulus for iHSP70 induction in a HSF1-dependent way.

Exercise is also known to induce eHSP70 release and accumulation into the circulation [107]. While the source of the eHSP70 during the exercise is still in debate, current data have suggested that hepatosplanchnic tissues should be involved [103]. Additionally, *α*1 adrenoreceptors in the liver seem to participate [118, 119] in a way that is dependent on exercise intensity, type, duration, and training status [120]. In addition, recent data suggests eHSP70 concentration increases once systemic temperature and sympathetic activity exceed minimum endogenous criteria and is likely to be modulated by large and rapid changes in core temperature [121]. However acute physical exercise is an inducer of eHSP70 release into the blood and exercise is not a factor of maintenance of high eHSP70 indefinitely, because chronic exercise (training) suppresses eHSP70 levels [101]. Hence, the expected deleterious effects of long-term exposure to high eHSP70 concentrations are never attained.

In response to eHSP70, macrophages can release IL-1*β*, IL-6, and TNF*α* [122], all cytokines involved in insulin resistance. Interestingly, in trained obese Zucker rats, the macrophage cytokine release profile in response to eHSP70 is changed. In this case, the macrophages from obese Zucker rats released less IL-1*β* and TNF*α* but more IL-6 than macrophages from lean animals, indicating that habitual exercise improved the release of proinflammatory cytokines by macrophages [122].

## 7. Concluding Remarks

Taken as a whole, the above findings clearly indicate that while iHSP70 is protective and anti-inflammatory being associated with normal insulin sensitivity, eHSP70 chronically produced in response to low-grade inflammation (but not to chronic exercise) is related to a proinflammatory response, decreased expression of iHSP70, and reduced insulin sensitivity. Therefore, we suggest that the ratio of HSP70 contents between the extra- and intracellular compartments may dictate the outcome of the inflammation and associated insulin resistance (Figure 1). Since exercise is able to modulate both eHSP70 and iHSP70, it is reasonable to predict that exercise is the most efficient and powerful tool currently available to normalize and/or maintain eHSP70/iHSP70 ratio at appropriate levels, thus preventing T2DM. The appropriate type, intensity, duration, and frequency of exercise that best fit each condition need to be determined.

The versatility of the HSP70 to induce different inflammation-related responses according to its location (intra- versus extracellular) places this protein as a master regulator for the fine-tuned control of the immune system: while iHSP70 induces inactivation of NF-*κ*B, eHSP70 induces the opposite effect. Thus, we suggest that the eHSP70/iHSP70 ratio may represent a better marker for the immunoinflammatory status of the whole body in exercise as well as in many types of diseases. Finally, we advocate that *R* values could be useful not only in assessing the course of therapeutic approaches in obesity-induced insulin insensitivity and diabetes, but also to evaluate inflammatory status in inflammation-related diseases (e.g., atherosclerosis and other cardiovascular diseases, rheumatoid arthritis, sepsis, and obesity-related nonalcoholic fatty liver disease) as well as in exercise training directed to immunosuppressed and cardiovascular patients. Hence, the above propositions may have an important diagnostic value for such patients, since “HSP70 status” determined through *R* values may be easily obtained from the ratio of extracellular (plasma) to intracellular (circulating blood leukocytes or mononuclear cells) HSP70 content from a blood sample.
